# The dry reforming of methane over fly ash modified with different content levels of MgO†

**DOI:** 10.1039/d1ra01381e

**Published:** 2021-04-14

**Authors:** Yufan Huang, Qi Li, Teng Zhao, Xiaofeng Zhu, Zijun Wang

**Affiliations:** School of Chemistry and Chemical Engineering, Shihezi University Shihezi Xinjiang 832003 PR China eavanh@163.com lqridge@163.com 1175828694@qq.com 318798309@qq.com wzj_tea@shzu.edu.cn; Key Laboratory for Green Processing of Chemical Engineering of Xinjiang Bingtuan, Key Laboratory of Materials-Oriented Chemical Engineering of Xinjiang Uygur Autonomous Region, Engineering Research Center of Materials-Oriented Chemical Engineering of Xinjiang Bingtuan Shihezi Xinjiang 832003 PR China

## Abstract

Large amounts of industrial waste fly ash (FA) have caused serious pollution to the environment. There are a few reports that this kind of material, with its good thermal stability, can be used as a catalyst support for high-temperature catalytic reactions, and it has a certain application space. Upon the alkali treatment of fly ash, its specific surface area is increased, and it has the potential to be a catalyst support. Using treated fly ash as the carrier, a nickel-based catalyst was prepared *via* a sol–gel method, and the catalytic performance changes of catalysts with different MgO content levels in the dry reforming of methane are discussed. Under the conditions of a space velocity of 1.8 × 10^4^ mL g^−1^ h^−1^ and a reaction temperature of 750 °C, in the presence of Ni/NaFA-M2 (M2 = 20 wt% MgO), the CH_4_ conversion rate can reach 84%, and it has good reaction stability. This will provide a way to use fly ash and carry out more research.

## Abstract 2

Fly ash is a kind of industrial waste, which is used as carrier to prepare nickel based catalyst. The flow chart of catalyst preparation is shown in the figure.

## Graphical abstract

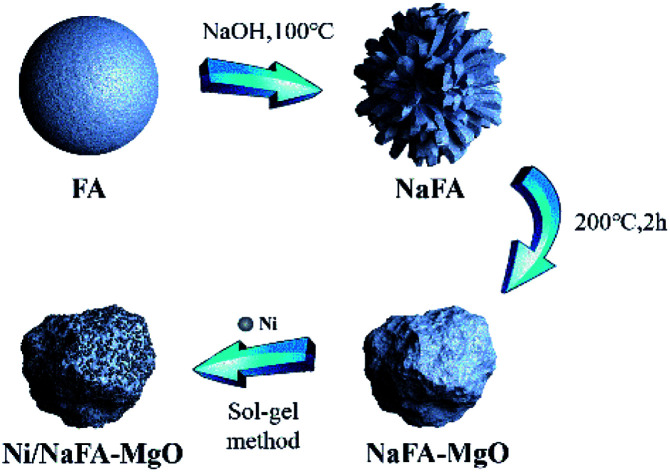

## 1. Introduction

Excessive coal combustion and industrial production have caused a large amount of greenhouse gas emissions and brought about the greenhouse effect, which has restricted the development of society and seriously endangered the living environment of human beings. At the same time, among greenhouse gases such as CO_2_, N_2_O, and CH_4_, CO_2_ accounts for 76% of the world's total greenhouse gases,^1^ and it has good stability and has been considered the most polluting gas. In this context, dry methane reforming (DRM) technology has become a research hotspot. This method can make two prevalent kinds of greenhouse gas, CO_2_ and CH_4_, react to generate clean energy in the form of H_2_ and CO. Compared with partial oxidation or steam reforming, the synthesis gas ratio of H_2_/CO is close to 1.^2^ It can be used in the Fischer–Tropsch synthesis reaction. Under appropriate conditions, it can synthesize liquid fuel mainly based on paraffinic hydrocarbons. To a certain extent, it can solve the problems of the energy crisis and environmental pollution caused by global warming.

The main reaction formula of methane dry reforming is

1 CO_2_ + CH_4_ → 2CO + 2H_2_, Δ*H*_298 K_ = +247 kJ mol^−1^,

but in addition to the main reaction, the following side reactions may also exist:^3^

Methane cracking reaction:

2 CH_4_ ⇔ C + 2H_2_, Δ*H*_298 K_ = +75 kJ mol^−1^,

Reverse water gas reaction:

3 CO_2_ + H_2_ ⇔ CO + H_2_O, Δ*H*_298 K_ = +41 kJ mol^−1^,

Carbon monoxide disproportionation reaction:

4 2CO ⇔ C + CO_2_, Δ*H*_298 K_ = −171 kJ mol^−1^,

The DRM reaction is an endothermic reaction (eqn (1)). In most cases, the reaction requires a temperature above 643 °C to achieve a high equilibrium of CO_2_ and CH_4_ conversion into H_2_ and CO synthesised gas.^4^ In this reaction, many studies have confirmed that the use of nickel as an active component to prepare a catalyst can give it good catalytic activity and stability. From an industrial point of view, compared with precious metals, low-cost, high-reserve nickel-based catalysts have more advantages. They have higher economic application prospects. However, under high-temperature reaction conditions, the active component nickel is prone to sintering and produces carbonaceous deposits. The carbon deposits may mainly come from the methane cracking reaction (eqn (2)) or the carbon monoxide disproportionation reaction (eqn (4)). This causes rapid deactivation of the catalyst. Therefore, various methods have been developed to improve the sintering resistance and carbon deposition resistance of the catalyst. Through the catalyst preparation method,^3,5–7^ active component loading content,^8^ carrier modification method,^9–11^ additives^12^ and other means affecting the catalyst structure, the active component particle size, catalyst pH and other aspects can be changed to improve the catalyst performance. The addition of alkali metals or alkaline earth metals such as MgO, CaO, or BaO to the catalyst can adjust the acidity and basicity of the catalyst, reduce the particle size of the active component and enhance the metal-support interaction (MSI), thereby effectively reducing the sintering of active components and inhibiting carbon deposition to a certain extent.^13–15^ Sogand Aghamohammadi *et al.*^16^ studied the influence of the sol–gel method and the conventional impregnation method on nickel-based catalysts in the dry reforming of methane. The results show that a catalyst NiO prepared by the sol–gel method has better dispersibility, and the catalyst has better catalytic activity and carbon deposition resistance. Therefore, we chose the sol–gel method as the catalyst preparation method.

Fly ash (FA) is an industrial waste discharged from coal-fired power plants. It is a mixture of crystals, glass and a small amount of unburned charcoal with a composite structure. The development and utilization of low-cost fly ash is a good choice for protecting the ecological environment and opening up new economic opportunities. Both SiO_2_ and Al_2_O_3_ are common catalyst supports. The initial activity of a catalyst prepared with SiO_2_ as the carrier is relatively low.^17^ Although a catalyst prepared with Al_2_O_3_ as the carrier has higher initial activity, the surface properties of Al_2_O_3_ are that it is acidic and has weak CO_2_ adsorption capacity, so the catalyst is prone to carbon deposition and deactivation.^18^ Fly ash is mainly composed of amorphous SiO_2_, Al_2_O_3_ and other oxides.^19,20^ The presence of a high content of SiO_2_ and Al_2_O_3_ gives fly ash good thermal stability, and it is used as a catalyst carrier, and is widely used in various reaction fields. Vikranth Volli *et al.*^21^ used fly ash as a carrier and added waste animal bone meal to synthesize a transesterification catalyst. Lei Gong *et al.*^22^ used fly ash as a carrier to synthesize a solid acid catalyst for the production of furfural from the hydrolysate of corn stover. Pramendra Gaurh *et al.*^23^ used fly ash to synthesize a low-cost natural catalyst. Shurong Wang *et al.*^24^ used fly-ash-supported nickel as a catalyst. The catalyst has good activity at 700 °C and the stability time can reach more than 10 hours. Therefore, fly ash has the potential to become a catalyst support for the methane dry reforming reaction.

In this study, in order to increase the specific surface area of fly ash and make it a suitable carrier for the DRM reaction, NaOH and HCl solutions were selected to activate and characterize fly ash. To improve the catalytic performance of the Ni based catalyst, alkali metal oxide MgO was added. It is reported that MgO can form a solid solution with NiO because its crystal structure is similar to NiO, so as to improve the sintering resistance of the catalyst. In addition, it has a good reduction temperature, increases the alkalinity of the catalyst, improves the adsorption of acidic CO_2_ by the catalyst, and can achieve the purpose of increasing the activity of the catalyst.^25–27^ By adding MgO and activated fly ash to the composite, loading the active component nickel, and using the sol–gel method to synthesize Ni/NaFA-MgO catalysts, the catalysts obtained under different MgO contents were extensively characterized and catalytic activity research conducted. This research may provide a new approach for FA treatment and develop a cheap and efficient methane dry reforming catalyst with potential industrial application value.

## 2. Experimental

### 2.1 Synthesis of MgO incorporated fly ash composite (FAMgO)

Fly ash (FA) was used in a control experiment, and different solutions were used to modify it. FA was placed in 2mol L^−1^ NaOH solution and 2mol L^−1^ HCl solution at a solid–liquid ratio of 1 : 10, and then the mixture was reacted at 100 °C for 24 hours. Then the FA was cooled to room temperature, filtered, washed until neutral and dried to obtain treated fly ash (NaFA). The final fly ash was then subjected to physical and chemical analysis. The chemical composition of fly ash is shown in Table 1. Appropriate products were selected for the follow-up experiments.

**Table 1:** Chemical compositions of fly ash samples

Constituent	Mass (%)		
	FA	FA-NaOH	FA-HCl
SiO_2_	52.75	44.50	59.62
Al2O_3_	31.98	31.02	32.09
Na_2_O	0.56	12.48	0.42
Fe_2_O_3_	4.60	4.09	2.31
CaO	4.40	4.40	1.21
K_2_O	2.07	0.55	2.04
TiO_2_	1.24	1.00	1.18
Specific surface area (m^2^ g^−1^)	2.4	59.5	18.5

1 g of urea was dissolved in 40 mL of deionized water and 1.5 g of magnesium acetate was dissolved in 50 mL of deionized water, respectively, and then the two solutions were mixed and stirred for 30 minutes. Subsequently, 0.9858 g of NaFA was added to the above mixed solution and stirred at room temperature for 5 hours, and then the mixed solution was stirred at 80 °C until it became a sol–gel. After drying overnight, it was calcined at 200 °C for 2 hours to finally obtain NaFA-30% MgO. According to the above method, NaFA-10% MgO and NaFA-20% MgO were prepared respectively as catalyst supports.

### 2.2 Catalyst preparation

**Fig. 1:**
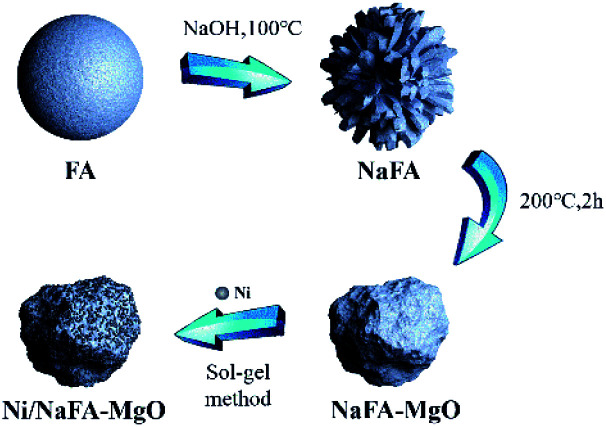
A catalyst-preparation flow chart.

Ni/NaFA-MgO catalysts modified with different proportions of MgO were prepared by the sol–gel method. The catalysts containing 10%, 20% and 30% MgO are referred to as Ni/NaFA-M1, Ni/NaFA-M2 and Ni/NaFA-M3, respectively. First, an appropriate amount of Ni(NO_3_)_2_·6H_2_O and citric acid was dissolved in a small amount of deionized water to prepare an Ni precursor. A certain amount of NaFA was added to 30 mL of deionized water, stirred at room temperature for 15 minutes, and then the mixture was added to the precursor solution, stirred at 80 °C, and a small amount of polyethylene glycol 400 added after 30 minutes. It was further stirred until it became a sol–gel, heated in an oven for 24 hours, and then calcinated at 750 °C for 4 hours to prepare the catalyst. The nickel loading was 12%. The preparation process is shown in Fig. 1.

### 2.3 Catalytic reaction tests

The performance test of the catalyst was carried out on a micro fixed-bed reactor. The test process was as follows: a certain amount of catalyst was weighed with a size of 40–60 mesh and mixed with 6 times the mass of quartz sand of the same mesh. Before the reaction, it was purged with N_2_ for 10 minutes, and then a 5 vol% H_2_–Ar mixed gas with a flow rate of 50 mL min^−1^ was used to reduce it at 800 °C for 2 hours. After the reduction, the temperature was changed to the required reaction temperature of 750 °C, and a mass flow meter was used to control the flow rates of CO_2_ and CH_4_ (CH_4_ : CO_2_ : N_2_ = 1 : 1 : 1). The reactant conversion rate and product distribution were tested at a space velocity of 1.8 × 10^4^ mL g^−1^ h^−1^ and 750 °C. A GC-9790 gas chromatograph was used for online analysis, and a TDX-01 packed column was used to separate H_2_, N_2_, CO, CO_2_ and CH_4_. The carrier gas was high-purity Ar, the flow rate was 30 mL min^−1^, and TCD detection was used.

## 3. Results and discussion

### 3.1 Physical and chemical characteristics of treated fly ash

**Fig. 2:**
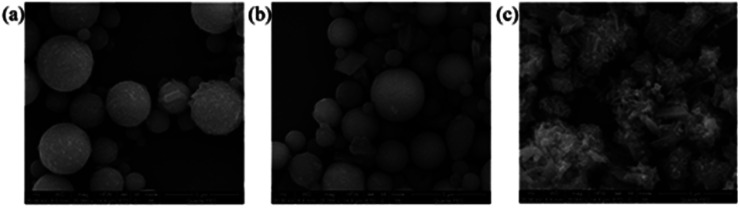
SEM images of fly ash: (a) untreated original fly ash, (b) HCl-treated fly ash, and (c) NaOH-treated fly ash.

**Fig. 3:**
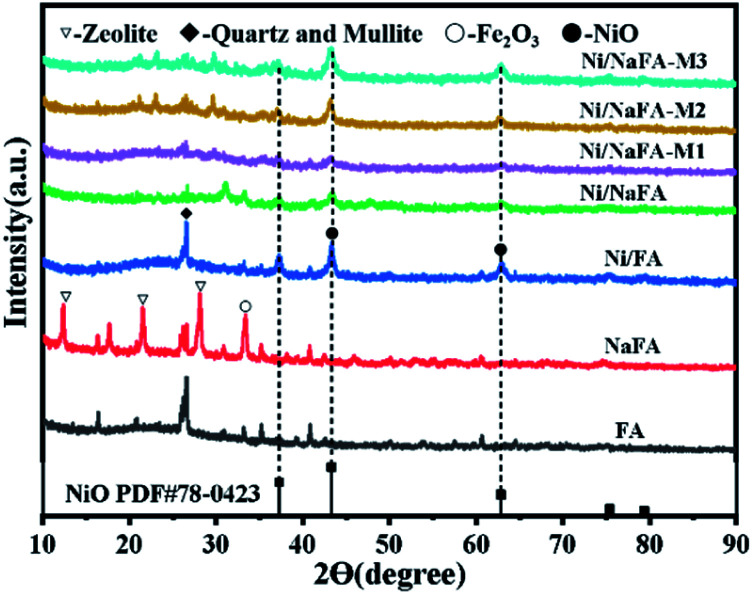
XRD patterns of fresh catalysts.

The chemical composition of the sample is shown in Table 1. The main components of fly ash are oxides of Si and Al, as well as various other oxides. It can be seen from Fig. 2 that after HCl treatment, the surface of the FA microspheres did not change significantly, while the specific surface area of FA increased. As shown in Table 1, after treating fly ash with HCl, the Fe_2_O_3_ content in FA decreased from 4.99% to 2.31%, while the CaO content decreased from 4.78% to 1.21%, resulting in a relative increase in SiO_2_ and Al_2_O_3_ content. The main reason for the decrease in Fe_2_O_3_ and CaO content is that the neutralization between Fe_2_O_3_, CaO and HCl leads to the dissolution of Fe_2_O_3_ and CaO. After alkali treatment, the main components of untreated fly ash FA and HCl-treated fly ash are similar, while the Na content in fly ash treated with NaOH is significantly increased. Since NaOH reacts with FA, SiO_2_ and Al_2_O_3_ to form silicate, it still contains a large amount of Na after washing NaFA to neutrality. In Fig. 2, it is found that NaOH solution affects the properties of FA by deforming the spherical shape and transforming its smooth surface into crystals of various shapes (such as flakes and rods). The flakes and rods on the FA surface are generated zeolite substances. This is confirmed in the XRD data of this experiment. As shown in Fig. 3, the appearance of Na_3.552_(Al_3.6_Si_12.4_O_32_)(H_2_O)_10.656_ P-type zeolite and Na_6_Al_6_Si_10_O_32_ sodium aluminosilicate is observed. The results are similar to those reported by P. Pengthamkeerati *et al.*,^28^ which indicates that fly ash can be transformed into zeolite materials and silicate materials under alkaline conditions. At the same time, the specific surface area of FA after alkali treatment has been significantly increased. This is the result of the conversion of fly ash into a zeolite-like structure under alkaline conditions.^28^

### 3.2 XRD

Fig. 3 shows the XRD patterns of fresh catalysts synthesized with different MgO composite amounts at 2*θ* = 10–90°. The catalysts containing 10%, 20% and 30% MgO are referred to as Ni/NaFA-M1, Ni/NaFA-M2 and Ni/NaFA-M3, respectively. In Fig. 3, we can clearly see the crystallization peak of NiO, indicating that NiO has been successfully loaded on NaFA. As we all know, as the content of MgO increases, Mg^2+^ will diffuse into the NiO lattice and move to a low angle according to Vegard's law. This can indicate that under the interaction of NiO and MgO, the two can form a solid solution to achieve the purpose of controlling Ni particle size.^29,30^ It can be seen from Table S1† that the particle size of Ni is smaller than that of Ni/NaFA after reduction at 800 °C after adding MgO. Furthermore, NiO(200) in Ni/NaFA-M1 appears at 43.0°, which is lower than the diffraction angle of pure NiO(200). This indicates that Mg^2+^ diffuses into the NiO lattice, so there may be “free” NiO or NiO-based NiO–MgO solid solution in Ni/NaFA-M1. At the same time, the position of the NiO(200) diffraction peak in Ni/NaFA-M2 and Ni/NaFA-M3 is also lower than that of NiO(200) at 43.2°, which proves that Ni^2+^ diffuses into the MgO lattice to form an MgO-based NiO–MgO solid solution.

### 3.3 FTIR

Fig. S1† shows the FTIR spectrum of the Ni/FA catalyst in the range of 400–4000 cm^−1^. In this range, at 3300–3600 cm^−1^ were observed the surface hydroxyl of Si–OH and the peaks produced by the H–O–H stretching vibration of the surface adsorbed water molecules.^31^ The absorption peak in the region of 1500–1750 cm^−1^ is related to the incompletely combusted carbon particles in the fly ash.^32^ The peak of the catalyst loaded with Ni disappears here, which may be because the catalyst is calcined at 750 °C. The grain disappeared. A strong absorption peak at 1002 cm^−1^ is caused by the strong asymmetric stretching vibration of Si–O–Si and Al–O–Si tetrahedrons due to SiO_2_ as the matrix, which indicates that Al has penetrated into Si–O–Si. A zeolite structure formed in the grid structure. At the same time, compared with untreated FA, the absorption peak of NaFA after alkali treatment shifted to a lower wavenumber direction. This may be due to the substitution of Na^+^ for the Si–O–Si and Al–O–Al in the depolymerized silica and alumina tetrahedrons. Some of the groups on the Al–O–Al chain affect their stretching vibration peaks, resulting in a peak shift phenomenon.^32^ The shift of the absorption peak of Ni/NaFA-MgO to a smaller wavenumber may be caused by the addition of Mg^2+^, which depolymerizes the silicate framework structure.^31^ The absorption peak observed at 468 cm^−1^ of the catalyst can be attributed to Ni–O and Mg–O. The absorption peaks of FA and NaFA at 441 cm^−1^ belong to the in-plane bending vibration of the Si–O bond. In NaFA, the peak shift may also be caused by the influence of Na^+^. In addition, the peak value in the 500–1000 cm^−1^ region may be related to alumina and mineral compounds.^26^

### 3.4 H_2_-TPR

**Fig. 4:**
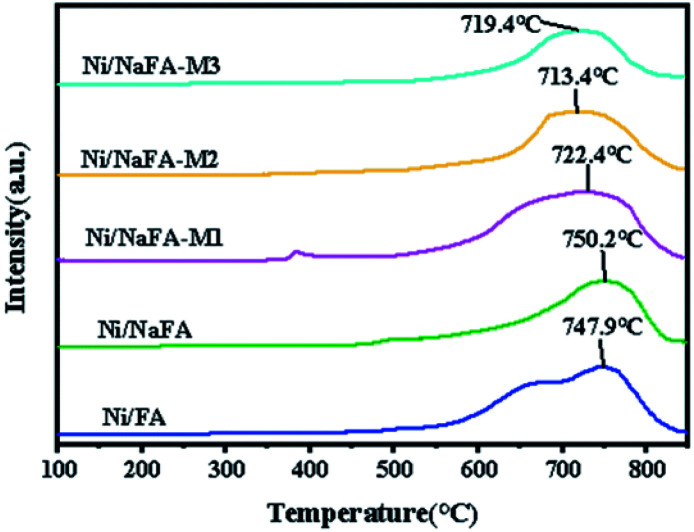
H_2_-TPR curves from the catalysts.

The H_2_-TPR characterization results of the catalyst are shown in Fig. 4. Ni/FA and Ni/NaFA have a high and broad peak at 500–850 °C, and the *T*_max_ for this peak is at around 747.9 °C, which is due to the conversion of Ni^2+^ to Ni element.^33^ After adding MgO, the catalyst has a broad single reduction peak between 600 °C and 850 °C, and the *T*_max_ are about 722.4 °C, 713.4 °C and 719.4 °C, respectively. Due to the interaction between NiO and MgO, the reduction temperature of the Ni/MgO catalyst is lower than that of other catalysts. The reduction peak of the catalyst usually moves to the low-temperature region,^17^ and the peak area is significantly reduced, and the reduction peak area of the catalyst increases with the addition of MgO. As the amount increases, the area gradually decreases, and the fraction of reducible Ni decreases with the increase in MgO content.^34–36^

### 3.5 BET

Table 2 summarizes the specific surface area, pore volume and average pore size of the catalyst. As can be seen, NaOH has an effect on the treatment of fly ash and the MgO content has an effect on the pore size distribution. After the addition of MgO, the specific surface area of the catalyst increased. The specific surface area and pore volume of Ni/NaFA-M2 increased from 12.3 m^2^ g^−1^ and 0.05 cm^3^ g^−1^ to 30.1 m^2^ g^−1^ and 0.10 cm^3^ g^−1^, respectively. This phenomenon is consistent with the report published by J. Ashok.^17^ When the MgO content increases to 30%, some MgO species participate in the formation of a non-reducible Ni–Mg-O_*x*_ solid solution, resulting in Ni/NaFA-M3, where the specific surface area and pore volume decreased to 20.9 m^2^ g^−1^ and 0.06 cm^3^ g^−1^, respectively.^37^

**Table 2:** Specific surface areas of catalyst samples

Catalyst	*S*_BET_ a (m^2^ g^−1^)	Pore volume^b^ (cm^3^ g^−1^)	Pore width^c^ (nm)
Ni/FA	12.3	0.05	16.6
Ni/NaFA	14.4	0.08	19.8
Ni/NaFA-M1	15.8	0.04	9.8
Ni/NaFA-M2	30.1	0.10	10.2
Ni/NaFA-M3	20.9	0.06	9.1

a Calculated using the BET equation.

b BJH desorption pore volume.

c BJH desorption average pore diameter.

### 3.6 XPS

**Fig. 5:**
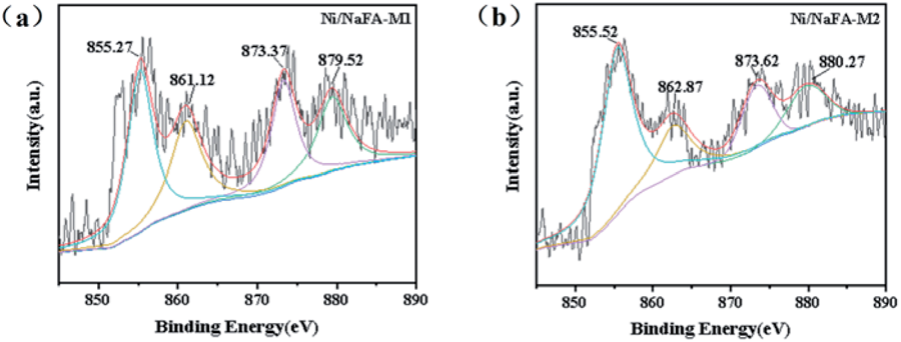
XPS analysis of spent catalysts: (a) Ni/NaFA-M1 and (b) Ni/NaFA-M2.

The XPS curves of the Ni/NaFA-M1 and Ni/NaFA-M2 spent catalysts are shown in Fig. 5. The chemical oxidation state of Ni metal on the surface of the spent catalysts was measured. Generally for all catalysts, the BE range of Ni 2p_1/2_ is 870–878 eV, and the BE range of Ni 2p_3/2_ is 850–860 eV.^38^ In Fig. 5(a) Ni/NaFA-M1, the spectrum at 855.27 eV represents the oxidation state of Ni, 861.12 eV is the satellite peak of Ni 2p, and the peak near 873.37 eV is the characteristic peak of Ni^2+^ species. The electron transfers from the MgO orbital to the unfilled Ni orbital charges. This electron transfer causes the interaction between MgO and Ni. As shown in Fig. 3, under the interaction of MgO and NiO, the position of the crystallization peak of NiO shifts, inhibiting the agglomeration of Ni and improving the activity of the catalyst.^27^ The eV value of the Ni/NaFA-M1 catalyst is lower than that of the Ni/NaFA-M2 catalyst. The higher the BE value, the stronger the interaction between the metal and the support, indicating that Ni/NaFA-M2 has better sintering resistance.^38^

### 3.7 TG

**Fig. 6:**
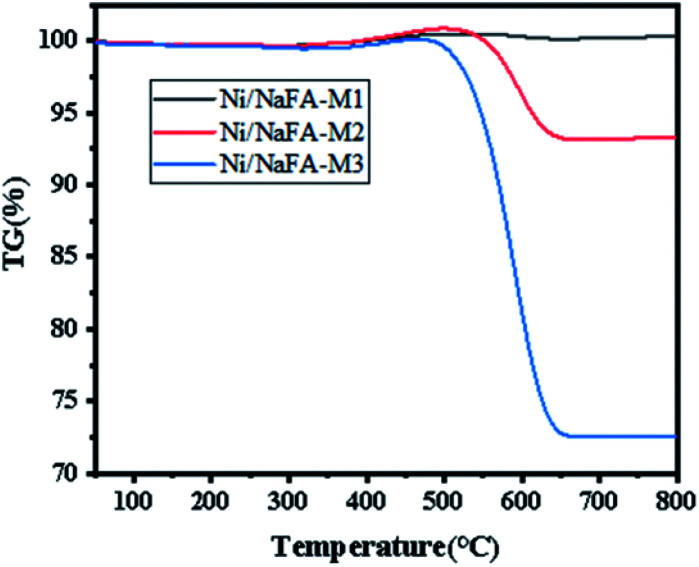
Thermogravimetry analysis of the catalysts.

Fig. 6 shows the TG analysis of spent catalysts: Ni/FA-M1, Ni/FA-M2 and Ni/FA-M3. The results show that Ni/FA-M1, Ni/FA-M2 and Ni/FA-M3 have a weight increase at 400–500 °C, which is mainly due to the mass increase caused by the reoxidation of the active metal Ni.^39,40^ As the temperature continues to rise, the removal of surface area carbon causes the weight of the sample to drop continuously. Among them, catalyst Ni/FA-M2 shows a weight loss of about 6.7%. Ni/FA-M3 has a relatively large weight loss (about 27.4%), which shows that Ni/FA-M3 has serious carbon deposits. However, Ni/FA-M1 shows no weight loss, indicating that adding 10% MgO to the catalyst gives it better resistance to carbon deposition. The comparison shows that Ni/FA-M1 has the strongest resistance to carbon deposition. With an increase in MgO content, the carbon deposition resistance of the catalyst is gradually weakened.^41^ It can be seen from the figure that Ni/FA-M2 and Ni/FA-M3 lose weight between 500 °C and 650 °C. This is due to the oxidation of filamentous carbon, resulting in a weight loss between 500 °C and 650 °C.^42^

### 3.8 TEM

**Fig. 7:**
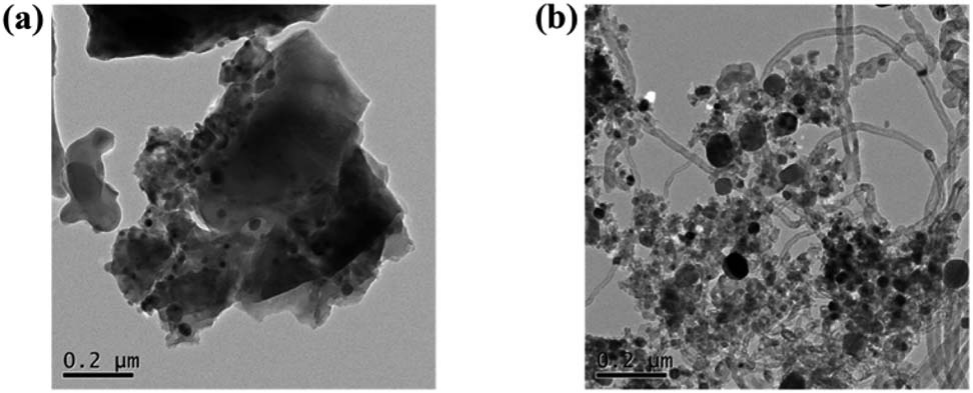
TEM analysis of spent catalysts after reaction at 750 °C and a CH_4_/CO_2_ of ratio 1 : 1: (a) Ni/NaFA-M1 and (b) Ni/NaFA-M2.

Fig. 7 shows the TEM images of spent catalysts Ni/NaFA-M1 and Ni/NaFA-M2. It can be clearly seen from the figure that there are carbon nanotubes (CNTs) on the surface of the spent catalyst Ni/NaFA-M2, while there are almost no carbon nanotubes on the surface of Ni/NaFA-M1, which confirms the results from the spent TG catalysts (Fig. 6). At the same time, it is found that adding a small amount of MgO helps to improve the carbon deposition resistance of the catalyst. Even if a large amount of carbon nanotube formation is detected in the sample with 20% MgO content, the stability of the catalyst is still very good, and the conversion rate of CH_4_ is maintained at 70% or more.

### 3.9 Catalytic stability tests

**Fig. 8:**
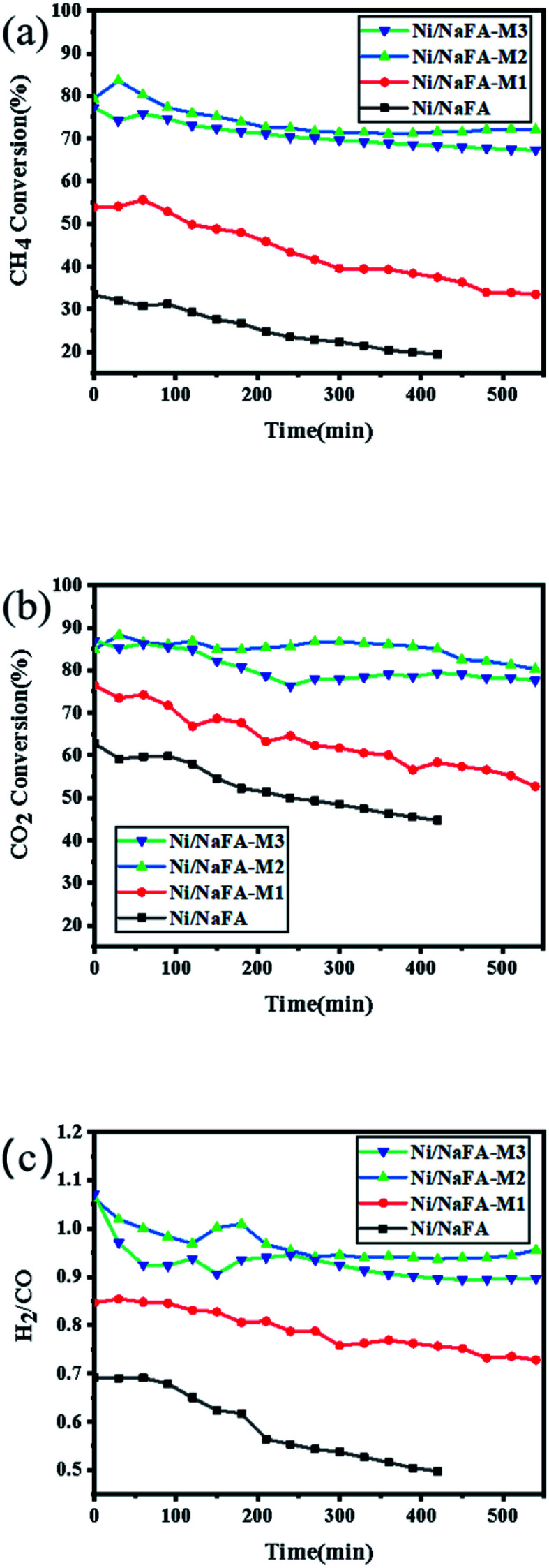
Catalyst stability at 750 °C for 9 h: (a) CH_4_ conversion rate, (b) CO_2_ conversion rate, and (c) H_2_/CO ratio.

Using methane and carbon dioxide as raw materials, the catalytic dry reforming reaction of methane was carried out at atmospheric pressure and 750 °C. The stability of Ni/NaFA was tested at 750 °C and 1.8 × 10^4^ mL g^−1^ h^−1^. Fig. 8 shows the effect of MgO on the stability of the Ni/NaFA catalyst. It can be seen that after the FA is modified with NaOH, the specific surface area of the catalyst increases, which has a catalytic effect on CH_4_ and CO_2_, and the content of MgO in the catalyst increases. The catalyst activity and stability also gradually increase. Among them, the catalyst with 20% MgO has the best catalytic performance and stability, which inhibits the reverse water gas reaction to a certain extent, so that the H_2_/CO of the reaction is basically maintained within 540 minutes around 0.95. This is because the basicity of MgO is similar to the crystal structure of NiO. The interaction between the two forms a solid solution, controls the size of Ni, and promotes the adsorption and activation of CO_2_, so that the intermediate product reacts with carbon on the catalyst surface to generate CO, thereby increasing the activity and stability of the catalyst.

## 4. Conclusions

In summary, a series of Ni/NaFA-MgO catalysts was successfully prepared using a sol–gel method. The results show that the use of NaOH to treat fly ash will significantly change the properties of fly ash, and the specific surface area is greatly improved. The sol–gel method was used with the Ni/NaFA catalyst to compound 10%, 20%, and 30% MgO and fly ash. The catalytic performance during the methane carbon dioxide reforming reaction was investigated, and the structure of the catalyst was characterized. The results show that Ni/NaFA-M1 has the lowest carbon deposition, but the catalytic activity is not high. The catalytic activities and stabilities of Ni/NaFA-M2 and Ni/NaFA-M3 are basically the same, and the carbon deposition of Ni/NaFA-M2 is far less than that of Ni/NaFA-M3, and the H_2_/CO ratio is closer to 1, showing the best results. XRD results confirmed that there is an interaction between NiO and MgO to form a solid solution, which improves the dispersion of NiO species in the catalyst and achieves the purpose of controlling the Ni particle size. Adding 20% MgO can increase the carbon deposition resistance of the catalyst, give higher catalytic activity, and better inhibit the reverse reaction of water vapor. The carrier has certain industrial application prospects in the methane dry reforming reaction.

## Conflicts of interest

There are no conflicts to declare.

## Supplementary Material

RA-011-D1RA01381E-s001
