# Supplementary material for: The dry reforming of methane over fly ash modified with different content levels of MgO
Source: RSC Adv. 2021 Apr 14;11(23):14154–60. doi: 10.1039/d1ra01381e (PMC8697673; doi:10.1039/d1ra01381e)
Supplement: RA-011-D1RA01381E-s001 [file RA-011-D1RA01381E-s001.pdf]

Electronic Supplementary Material (ESI) for RSC Advances.
This journal is © The Royal Society of Chemistry 2021

Supporting Information

for

Dry reforming of methane over fly ash modified with different content of MgO

*Yufan Huang[a,b], Qi Li[a,b], Teng Zhao[a,b], Xiaofeng Zhu[a,b], Zijun Wang[a,b]**

[a] School of Chemistry and Chemical Engineering, Shihezi University, Shihezi, Xinjiang 832003, PR China.

[b] Key Laboratory for Green Processing of Chemical Engineering of Xinjiang Bingtuan/Key Laboratory of Materials-Oriented Chemical Engineering of Xinjiang Uygur Autonomous Region/Engineering Research Center of Materials-Oriented Chemical Engineering of Xinjiang Bingtuan, Shihezi, Xinjiang 832003, PR China.

***Corresponding Author:** Zijun Wang

Address: Beisi Road, Shihezi City, Xinjiang, 832003, P. R. China.

Tel: 15699322089

E-mail Address: wzj_tea@shzu.edu.cn

Table S1. NiO(200) XRD diffraction angle and Ni particle size of Ni/FA samples.

Sample name	NiO particle size by XRD	Reduction catalyst Ni particle size by XRD	Used catalyst Ni particle size by XRD	XRD angle (2Θ) (degree) NiO(200)
NiO	-	-	-	43.2
Ni/FA	18nm	30nm	31nm	43.3
Ni/NaFA	14nm	28nm	28nm	43.3
Ni/NaFA-	11nm	18nm	23nm	43.0

M1				
Ni/NaFA-M2	16nm	21nm	30nm	43.1
Ni/NaFA-M3	12nm	23nm	27nm	43.2

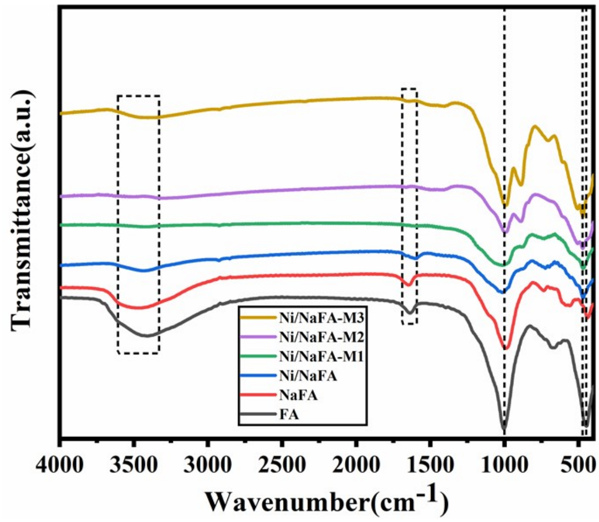

Figure S1. FTIR spectra of catalysts with different MgO content.
